# Molecular architectonics of DNA for functional nanoarchitectures

**DOI:** 10.3762/bjnano.11.11

**Published:** 2020-01-09

**Authors:** Debasis Ghosh, Lakshmi P Datta, Thimmaiah Govindaraju

**Affiliations:** 1 Bioorganic Chemistry Laboratory, New Chemistry Unit and The School of Advanced Materials (SAMat), Jawaharlal Nehru Centre for Advanced Scientific Research, Jakkur P. O., Bengaluru 560064, Karnataka, India

**Keywords:** DNA nanotechnology, functional DNA nanoarchitectonics, functional small molecules, molecular architectonics, nucleic acids, templated coassembly

## Abstract

DNA is the key biomolecule central to almost all processes in living organisms. The eccentric idea of utilizing DNA as a material building block in molecular and structural engineering led to the creation of numerous molecular-assembly systems and materials at the nanoscale. The molecular structure of DNA is believed to have evolved over billions of years, with structure and stability optimizations that allow life forms to sustain through the storage and transmission of genetic information with fidelity. The nanoscale structural characteristics of DNA (2 nm thickness and ca. 40–50 nm persistence length) have inspired the creation of numerous functional patterns and architectures through noncovalent conventional and unconventional base pairings as well as through mutual templating-interactions with small organic molecules and metal ions. The recent advancements in structural DNA nanotechnology allowed researchers to design new DNA-based functional materials with chemical and biological properties distinct from their parent components. The modulation of structural and functional properties of hybrid DNA ensembles of small functional molecules (SFMs) and short oligonucleotides by adapting the principles of molecular architectonics enabled the creation of novel DNA nanoarchitectures with potential applications, which has been termed as templated DNA nanotechnology or functional DNA nanoarchitectonics. This review highlights the molecular architectonics-guided design principles and applications of the derived DNA nanoarchitectures. The advantages and ability of functional DNA nanoarchitectonics to overcome the trivial drawbacks of classical DNA nanotechnology to fulfill realistic and practical applications are highlighted, and an outlook on future developments is presented.

## Review

### Introduction

**Figure 1:**
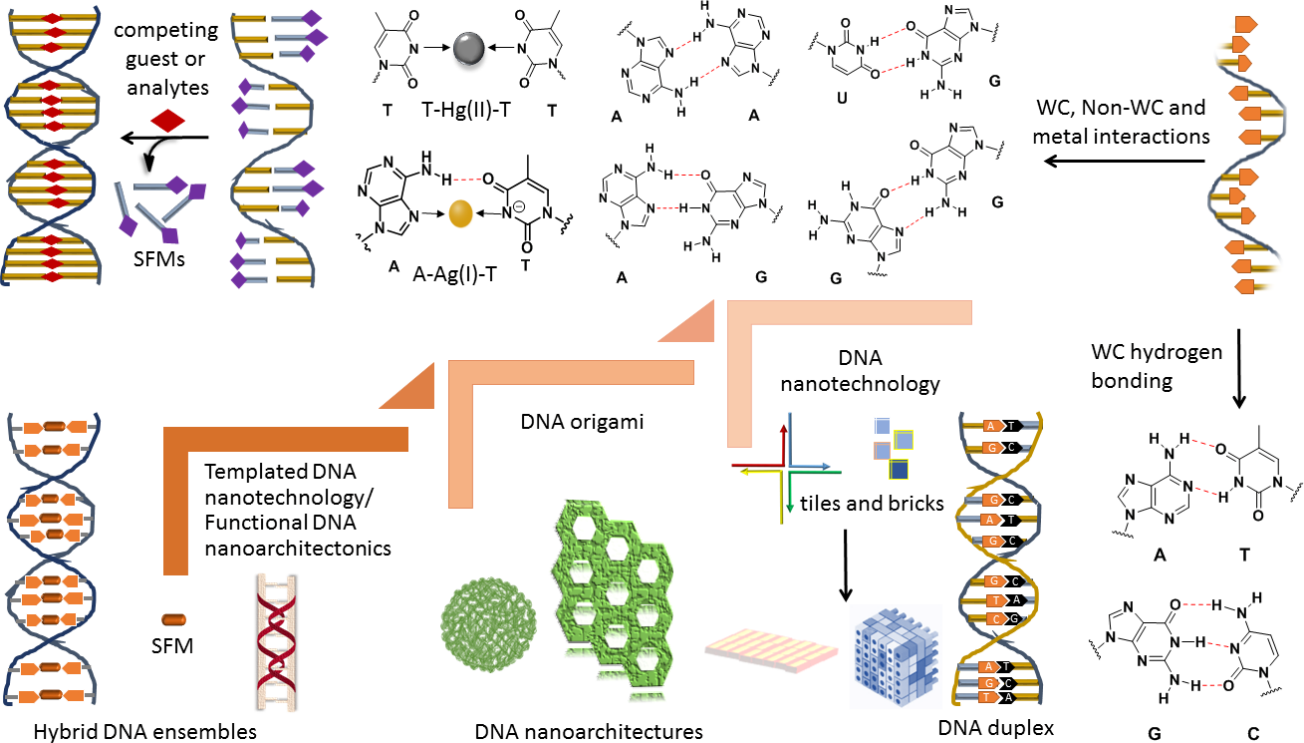
Illustration of conventional (WC) and unconventional (non-WC) hydrogen bonding interactions between the nucleobases to form canonical (DNA duplex) and noncanonical hybrid DNA ensembles through the assembly of DNA or DNA with SFMs/metal ions. The brick image has been adopted with permission from [17], copyright 2012 American Association for the Advancement of Science.

The development of functional molecular systems and materials on a nanoscale through custom design and engineering of molecular organization is a highly attractive concept in materials science [1–2]. Exploitation of biomolecules and their in-built information for molecular recognition to engineer ordered assemblies and coassemblies of SFMs is termed as molecular architectonics [3–4]. The construction of molecular architectures through the controlled assembly of designed molecular units with fascinating properties and functions is central to all materials and bioengineering processes [1–4]. The use of biomolecules or synthetic systems with biomolecular components is capable of aiding the judicious regulation of molecular assembly parameters and properties to construct novel functional architectures in the scheme of molecular architectonics [1–2]. Among all biomolecules, DNA, with a well-defined structure, is the epitome of molecular recognition and a robust system for molecular and materials engineering. The molecular stability, predictable sequence specificity, molecular recognition properties, and the formation of regular and defined structures of DNA made it possible to custom the design and to engineer a range of molecular architectures [5–9]. In DNA, two polydeoxyoligonucleotides (single-stranded DNA, ssDNA) are held together by complementary or conventional Watson–Crick (WC) base pairing interactions (Figure 1). In WC base pairing interactions, adenine (A) and thymine (T) form a doubly hydrogen-bonded base pair (A=T), while guanine (G) and cytosine (C) form a triply hydrogen-bonded base pair (G≡C) [10]. The hydrogen bonding-mediated base pairing geometry is conditional on the conformation of the glycoside bonds and interactive hydrogen bonding sites. Apart from WC hydrogen bonding, unconventional hydrogen bonding, electrostatic, and metal ion interactions play a significant role in the formation of noncanonical DNA architectures (Figure 1) [5]. The noncanonical hydrogen bonding interactions are responsible for the formation of a range of higher-ordered DNA structures. In particular, the double-stranded DNA duplex is a perfect nanoscale molecular architecture with a 2 nm thick rigid structure and a persistence length of ca. 40–50 nm. Moreover, ssDNA sequences can be used as molecular glue to construct diverse and well-defined nanoarchitectures. In this context, Seeman and co-workers introduced the disruptive idea of using DNA as a molecular building block to design and construct nanosystems and materials, which paved the way for the celebrated area of DNA nanotechnology [11]. Nevertheless, the nanoscale structural features of DNA have inspired the design of diverse functional architectures utilizing both conventional and unconventional base pairing along with the mutually templating interactions of SFMs and metal ions [12]. The field of DNA nanotechnology has evolved over the years from using DNA tiles and blocks to employing SFMs and their assemblies as templates to control the molecular organization on the nanoscale to generate complex DNA architectures or origami and hybrid ensembles, respectively, through the judicious exploitation of conventional and nonconventional base pairing interactions [12–13].

The field of DNA nanotechnology was further advanced by the DNA origami concept introduced by Rothemund and co-workers [14–15]. Intertwining and congregation of more than one DNA strand to produce DNA tiles or bricks, which staple in a programmed fashion to form crystalline assembly structures with well-defined geometries, constitute the guiding principles of specific nucleobase pairing-driven DNA nanostructures (DNA nanotechnology) [16–17]. In other words, DNA origami involves the programmed folding of long DNA sequences to generate defined but complex shapes on the nanoscale. Despite the expeditious advancements in the field of DNA nanotechnology, utilization of long DNA sequences, complex computer-based design strategies, reproducibility, and the high cost involved in the entire process have become the limiting factors in the realization of practical applications. In this context, the emerging field of SFM-templated or mutually templated DNA nanotechnology (functional DNA nanoarchitectonics) is considered the state-of-art to construct hybrid DNA nanoarchitectures with assured functional properties and practical applications [12–13,18–21]. In functional DNA nanoarchitectonics, short oligonucleotides (ssDNA) with sequences of less than persistence length (ca. 40–50 nucleotides) are co-organized with SFMs or their assemblies to generate hybrid DNA ensembles [18,20]. The short oligonucleotides are inexpensive to synthesize in-house or commercially available, and their coassembly with SFMs assures to generate novel nanoarchitectures with functional properties and applications. Although the field of classical DNA nanotechnology exploited the supramolecular bottom-up self-assembly, the functional features can be integrated through mutually templated coassembly of SFMs and short ssDNA sequences in the emerging field of functional DNA nanoarchitectonics [22]. Remarkably, engineering molecular coassemblies of SFMs and ssDNA to generate functional DNA nanoarchitectures represents a seamless relationship between the molecular architectonics and nanoarchitectonics. The field of nanoarchitectonics has been introduced and pioneered by Aono and Ariga at NIMS, Japan, who reported numerous self-assembly approaches to construct a range of nanoarchitectures [23–30].

The understanding and controlling of noncovalent interactions on the molecular level to engineer the assembly and coassembly of molecular components is a challenging task. Therefore, molecular architectonics of biomolecules with designer SFMs is an interesting and reliable approach wherein biomolecules with in-built information for molecular recognition guide the functional assembly and coassembly of SFMs. In essence, the molecular architectonics of DNA with SFMs to construct nanoarchitectures covers molecules to (nano)materials to functional applications. Typically, SFMs are suitably functionalized functional molecules with excellent optoelectronic properties that undergo π-stacking and support the co-organization of oligonucleotides to form hybrid DNA ensembles [18,20]. These hybrid DNA ensembles can be employed for a range of applications in the fields of materials science, nanotechnology, sensors, molecular or nanoelectronics, diagnostics, drug delivery, and biomedical sciences.

The remarkable molecular fidelity and sequence-specific molecular recognition make DNA the ideal candidate in the scheme of molecular architectonics to design and construct functional DNA nanoarchitectures. In this review, we attempted to cover the molecular architectonics of DNA, which comprises programmed self-assembly (DNA nanotechnology) and coassembly (templated DNA nanotechnology/functional DNA nanoarchitectonics) to produce diverse molecular and nanoarchitectures. The functional DNA nanoarchitectonics, encompassing the formation of functional hybrid DNA ensembles through coassembly of organic molecules (SFMs) and short oligonucleotides, is envisioned to overcome the limitations associated with classical DNA nanotechnology to realize practical applications [13]. The various design approaches of DNA self-assembly and coassembly that have been utilized to form novel nanostructures with a range of applications, from materials to biomedicine, are covered. The judicious exploitation of canonical and noncanonical base pairing interactions supported by various other noncovalent interactions for the creation of molecular and nanoarchitectures of DNA are highlighted. Overall, the aim of this article was to provide a brief overview on the molecular architectonics of DNA with respect to the historical perspective, the evolution of the celebrated area of DNA nanotechnology, and recent advancements in the form of functional DNA nanoarchitectonics to realize practical applications.

### Classical DNA nanotechnology: programmed molecular self-assembly of DNA

The use of DNA as a building block for the construction of nanomaterials typically involves exploitation of the WC base pairing and predictability of the structural outcome owing to sequence specificity [31–32]. The DNA hybridization through WC base pairing (A=T and G≡C) effectively facilitates the programmability of the molecular self-assembly of DNA. In a standard WC base pairing-driven assembly, two complementary DNA strands anneal together to form a duplex structure. In a DNA hybridization process, the oligonucleotides with complementary base sequences are dissolved in a buffer solution and subjected to annealing, which involves a cycle of heating the solution followed by cooling [31]. Seeman and co-workers envisioned the construction of 3D nanoarchitectures by utilizing DNA as a structural building block and hybridization (base pairing) as the ‘glue’ [33–34]. The initial attempts with ssDNA were compromised due to the lack of rigidity within ssDNA molecular systems. However, the higher-order double-stranded DNA systems exhibited higher rigidity as compared to ssDNA. The selection of the correct base pairing sequence and order enabled the assembly of DNA with balanced rigidity and flexibility within the nanomaterial systems. The maintenance of an exact stoichiometry and long annealing period were the major criteria to achieve defect-free architectures from long DNA sequences. Rothemund, Yan, and co-workers introduced the concept of assembling so-called scaffold and staple strands to construct functional geometric architectures [15,35]. In this process of DNA assembly, the long scaffold strand can adapt different geometries upon interaction with multiple staple strands. The advantages of this concept are that the preservation of exact stoichiometric ratios of ssDNA sequences is not necessary and that the assembly can be performed faster. The introduction of staple strands to the scaffold initiates the local folding, and further addition of multiple staple strands leads to strand displacement, thereby healing the mismatches in long DNA scaffold strands.

**Figure 2:**
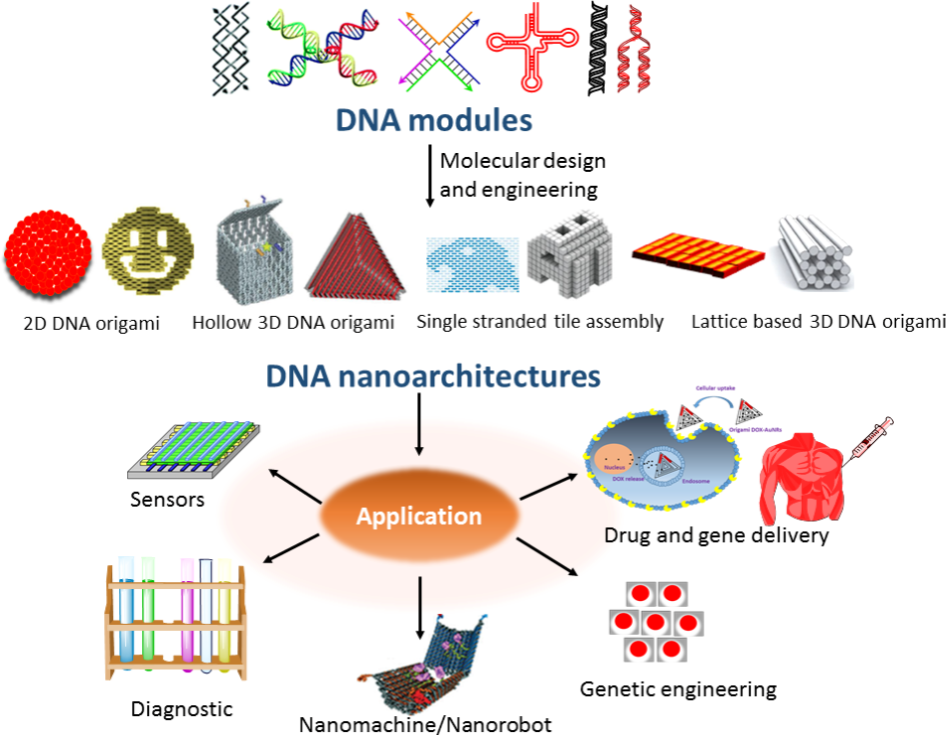
Molecular design and engineering of DNA nanoarchitectures using different types of DNA modules. The 2D DNA origami, hollow 3D DNA origami, single-stranded tile assembly, and lattice structures are adopted with permission from [37], copyright 2018 Wiley and Sons. The DNA double-crossover foundation and holiday junction structure have been reproduced with permission from [8], copyright 2016 Royal Society of Chemistry, [34], copyright 1993 American Chemical Society. The DNA nanorobot structure has been adopted with permission from [9], copyright 2012 American Association for the Advancement of Science. The first image on the left side of drug and gene delivery has been adopted with permission from [53], copyright 2012 American Chemical Society.

The sticky end cohesion method to create nanostructured ensembles is a radically different concept for DNA assembly [36]. The partial complementary feature of DNA used in this approach results in single-stranded overhang regions, so-called sticky ends. The binding interaction between two different but complementary sticky ends guides the sequential assembly of building blocks. The weak interactions, viz., hydrogen bonding and electrostatic interactions, act as driving forces for the assembly of building blocks to form ordered structures of DNA. Further, the self-assembly can be regulated by varying the extent of base pairing in double-stranded DNA or overhang regions of the building blocks. DNA nanotechnology has hugely benefitted from the recombinant DNA technology, which enabled the construction of complex architectonics through DNA origami [37]. The self-recognition characteristics of DNA allows the development of a wide array of DNA-based nanoarchitectures by employing an array of designer sequences and motifs. In the earliest designs, the Holliday junction structure of DNA was used to create nanostructured DNA materials [38–39]. The structural analysis of the self-assembled nanomaterials synthesized using junctional building blocks showed a compromised stability due to the sequence symmetry. The elimination of sequence symmetry is one of the most important criteria in DNA nanotechnology [40–41]. Exclusion of symmetry is equally important in DNA-based sequence and motif design. In the design of DNA nanomotifs, double-crossover tiles are designed specifically from ssDNA sequences. These double-crossover tiles have paved the way for the programmable construction of robust DNA nanoarchitectures with well-defined structural and functional properties. Moreover, the programed assembly of both single-stranded and double-stranded DNA can lead to the generation of a variety of nanoarchitectures with potential biological applications (Figure 2).

### Diversity in DNA nanoarchitectures

The structural diversity of DNA nanoarchitectures is the most attractive feature of DNA nanotechnology. From the application perspective, DNA nanotechnology can be divided into static and dynamic categories [42–43]. In static DNA nanotechnology, the DNA strands are immobilized within particular geometries, patterns, or crystals that facilitate the positional assembly of nanoparticles along the DNA nanostructures [42]. The dynamic DNA nanotechnology includes DNA nanomachines with potential applications ranging from sensing to delivery and robotics [43]. Inspired by the complementary base pairing-directed DNA hybridization, 1D, 2D, and 3D DNA nanoarchitectures can be constructed through three major methods: i) DNA tile-based construction, ii) DNA origami, and iii) nanoparticle-templated procedures. The tile-based method relies on multiple crossover junctions, which utilize a small number of short DNA sequences and impart enough rigidity to assemble nanoarchitectures. The designed short DNA strands initiate the formation of double- and triple-crossover building block structures, which are further utilized to make diverse architectures through a combinatorial approach. Mao and co-workers exploited the tile-based strategy for the construction of three-point star motifs using three different DNA sequences [44]. In this design, each arm corresponded to a double-crossover motif and its assembly led to the formation of 3D wireframe polyhedrons, depending on the concentration and curvature of the motifs. The curvature and flexibility could be modulated through incorporation of hairpin loops. Zhang and co-workers reported new strategies for the design of DNA wireframe nanostructures wherein the single-stranded tile (SST) method facilitated the formation of tubular structures of variable dimensions [45].

As discussed earlier, the pioneering work by Rothemund and others opened the era of DNA origami that aided the construction of unique and most complex architectures [46]. The rectangular DNA origami was gaining momentum in recent times, which involved studies on molecular engineering of DNA using rectangular tiles connected via dangling DNA strands [47–48]. Yan and co-workers reported the construction of rectangular DNA origami nanoarchitectures in which the parallel strands were assembled in a zigzag fashion along the DNA helical axis [46]. Endo and co-workers paved the way for creating X-shaped, Y-shaped, and asterisk-shaped structures using 2D and 3D DNA origami tiles [49–50]. The groups of Endo and Sungiyama showed that 2D origami architectures could be used as building blocks for the construction of 3D DNA origami [50]. Apart from creating a wide range of architectural shapes, DNA origami is anticipated to have possible applications in the fields of biosciences, the design of protein scaffolds, and plasmonics [51–53]. Shih and co-workers utilized DNA origami nanotechnology for the structural determination of plasma membrane proteins [54]. They reported the construction of detergent-resistant 0.8 µm-long liquid crystalline DNA nanotubes organized from a 7.3 kb scaffold strand and >170 short oligonucleotide-long staple strands. The liquid crystalline matrices of six helix DNA origami bundles induced a weak alignment of proteins within the plasma membrane. The solubilization and weak alignment of proteins within plasma membrane are crucial to measure the residual dipolar couplings (RDCs), and the RDCs are crucial to obtain NMR structural information on membrane-bound proteins. As reported, DNA origami-based liquid crystalline media can overcome detergent-related compatibility problems to accurately measure RDCs.

DNA nanomachines or nanorobots are highly innovative and advanced versions of DNA-based molecular designs, which are intended to act and perform as machines with respect to internal or external stimuli. The pioneering design and development of DNA-based molecular machines was introduced by Seeman and co-workers in 1999 [55]. In this early and seminal work, it was shown that branched motifs of DNA can be exploited as switchable mechanical machines. DNA double-crossover (DX) tiles were used to fabricate the molecular devices. The crossover points of the tiles were separated by a helical turn, which triggered the switchable motion of the device through B-to-Z-form transition, and the relative changes in position and transformation were monitored by the fluorescence resonance energy transfer (FRET) technique. Zhao and co-workers reported the design and construction of a DNA origami-based nanorobot for the cargo delivery of payloads into cancer cells [56]. The autonomous DNA nanorobot was constructed using a nucleolin-binding DNA aptamer and was loaded with thrombin protease. The nucleolin protein was overexpressed in tumor-associated endothelial cells, which triggered the mechanical opening of the DNA nanomachine, followed by the release of the cargo protease from the inner cavity to the targeted area. In vivo studies in mice demonstrated that the intravenously injected nanorobot could effectively deliver thrombin to tumor-associated blood vessels. The targeted delivery and nonimmunogenicity of the nanorobot made it a promising candidate for drug delivery in cancer therapeutics. The group of Krishnan reported the construction of a DNA nanodevice to quantitatively determine the activity and location of chloride ion channels and transport under pH stimuli [57]. In another work, they reported the construction of a DNA-based reporter nanomachine for quantitative imaging of lysosome [58]. This two-ion measurement (2-IM) method could image both pH and chloride ion variations in lysosomes. The 2-IM analysis was conducted on primary skin fibroblast cells derived from healthy and Neiman–Pick-diseased patients. The results showed significant differences in the lysosomal population in cells between the diseased and the healthy state. The group of Andersen reported a DNA nanobox with controllable lid, and its dynamic nature potentially facilitated the stimuli-responsive release of cargo molecules into the targeted area [59]. In another interesting design, DNA origami nanorobots were implanted into living systems and executed DNA-based biocomputing via dynamic cell-associated interactions [60]. An alteration of the physiological pH can be indicative of a diseased condition, and therefore monitoring physiological pH values with high sensitivity is required. Our group designed a molecular beacon (LMB) DNA device appended with a FRET pair as pH sensing probe in cells [19]. The remarkable feature of the LMB probe was the structural transition from a closed (molecular beacon) state to an open (A-motif) state in a pH-responsive manner within artificial vesicles and living cells. The DNA device was made up of 24 nucleobases, of which 12 adenine nucleobases were present within the loop region, and a closed molecular beacon structure was formed *via* two stretches of five complementary base pairs. Cy3 and Cy5 dyes acted as donor–acceptor FRET pair systems that were ligated at the 5' and 3' end of the duplex stem structure, respectively. Under normal physiological conditions, the closed hairpin structure of the LMB probe facilitated juxtaposition of the two dyes, followed by efficient FRET. In acidic pH, the N1 of adenine became protonated, which triggered the structural transition of the LMB device from a closed to an open state through reverse Hoogsteen base pairing and electrostatic interactions. The pH-responsive structural transition of LMB from a closed to an open state altered the FRET response, which was exploited for sensing of acidic pH (3–5.5) with a low step size (0.2–0.3) within synthetic vesicles that mimicked the intracellular environment. The in cellulo study in HeLa cells demonstrated the efficient cellular uptake of the DNA device without the need for a vector and provided efficient sensing of changes in the intracellular acidic pH value. In recent years, DNA thin film-based biosensors received significant interest for the detection of biologically relevant analytes, such has forensic samples [61–62]. The design of active electrochemical DNA sensors involves critical optimization of the sensor platforms. The length of the target oligonucleotide sequence and the selective use of dopants significantly dominate the sensing efficacy [63]. In this context, electrochemical DNA sensors were developed by noncovalent layer-by-layer assemblies of phenothiazine dyes and DNA for the detection of damaged DNA [64]. Apart from biological samples, the identification of volatile organic compounds is another important field gaining the attention of researchers. Hairpin DNA and peptide sequences were integrated in a sensor design strategy to develop an optoelectronic nose for the selective detection of volatile organic components [65].

### DNA tetrahedron nanostructures

**Figure 3:**
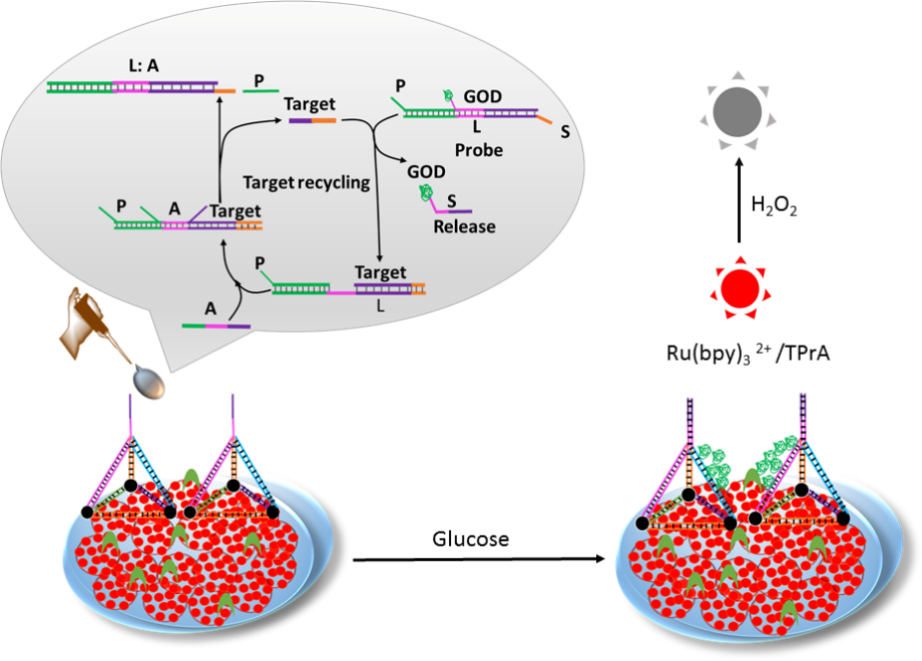
Schematic representation of DNA tetrahedron-based electroluminescence biosensor platforms. The image has been adapted with permission from [69], copyright 2017 American Chemical Society.

The structural analogy to virus particles makes DNA polyhedrons highly appealing architectures with biomimetic functional relevance. Among all polyhedrons, tetrahedrons are the simplest architectural scaffolds to construct and modulate structural and functional patterns. The tetrahedron with regular edges and acmes is a perfect architectural shape for the construction of DNA nanoarchitectures. The virus-mimetic feature of the DNA tetrahedron accounts for the facile cellular uptake via a caveolin-dependent pathway, while the rigid and sharp-edged features bestow the thermal and enzymatic stability. The construction of a DNA tetrahedron was first attempted by Turberfield and co-workers, wherein short oligonucleotide sequences were used for the bottom-up assembly process [66]. In addition to short oligonucleotide sequences, DNA tiles were also used to build DNA tetrahedrons. In another approach, DNA tetrahedron cages were prepared for efficient cellular uptake and imaging of live cells [67]. Human embryonic kidney (HEK) cells were cultured with a range of fluorescently tagged DNA tetrahedrons, and the subcellular localization was monitored. An organelle-staining study indicated the localization of the tetrahedrons within the cytoplasm and demonstrated efficient cellular uptake of DNA tetrahedrons without the need for any transfection agents or procedures. One of the advantages of DNA tetrahedrons is that the edges can be covalently modified with several active functionalities or biomolecules. The groups of Shangguan and Tan reported on the biofunctionalization of metal nanoparticles using aptamer-appended DNA tetrahedron nanostructures [68]. The aptamer-appended tetrahedron structures were constructed using three 55 nucleotide-long carboxylic acid-linked DNA strands and a tumor-targeting 87 nucleotide-long aptamer. The carboxylic acid groups of the DNA tetrahedron facilitated the interaction with oleic acid-coated iron oxide nanoparticles via a ligand exchange reaction. The aptamer–DNA tetrahedron-functionalized iron oxide nanoparticle system was capable of selectively targeting the cancer cells and, potentially, to act as an MRI contrast agent. The programmability of the DNA tetrahedrons provided an opportunity to conjugate other functional nucleic acid sequences, viz., DNA, siRNA, or DNAzymes, to serve as potential diagnostic or therapeutic (theranostic) nanoagents. Xu and co-workers demonstrated the derivation of a DNA tetrahedral electroluminescence (ECL) biosensor probe for a functional biosensing assay (Figure 3) [69]. The ECL biosensor platform was constructed based on a DNA tetrahedral scaffold embedded with Ru(bpy)_3_^2+^-conjugated silica nanoparticles. The DNA tetrahedron geometry acted as a capture DNA that repelled the nonspecific DNA entanglement along the ECL platform and stimulated the hybridization of glucose oxidase (GOD) enzyme-conjugated DNA (GOD-S). In a programmable cyclic amplification pathway, the target DNA triggered the release of GOD-S that catalyzed glucose to form hydrogen peroxide, followed by changing the ECL signal. The ECL sensing solution was made up of tripropylamine (TPrA) and glucose (10 mM). The concentration of target DNA could easily be assessed by quantifying the ECL quenching via formation of hydrogen peroxide. Kim and co-workers developed an innovative approach of intercalation of the anticancer drug doxorubicin within the DNA tetrahedron that showed improved therapeutic efficacy in drug-resistant breast cancer cells [70]. The doxorubicin-encapsulated DNA tetrahedron system exhibited enhanced cellular uptake and outflanked the drug efflux process in multidrug-resistant cancer cells. The biocompatible features, natural degradability, and low immunogenicity made the DNA tetrahedron a potential carrier platform for the delivery of drug cargos. Lee and co-workers showed that self-assembled DNA tetrahedron nanoarchitectures with narrow size distribution could deliver siRNA into tumor cells [71]. The programmable DNA strands were functionalized with tumor-targeting folate ligands. The nanoarchitecture consisted of six DNA strands having a total of 186 WC base pairs. The complementary overhang regions at the 3′ ends self-assembled to form tetrahedron nanoarchitectures. The edges of the tetrahedron were found to be thirty base pairs long and the height was ≈8 nm. The middle of each edge consisted of a nick with an overhang region whose sequence was exactly complementary to the siRNA sequence, and, typically, six siRNAs could be annealed with the tetrahedron unit. To ascertain the in vivo transfection efficiency of the siRNA-loaded DNA tetrahedron nanoarchitecture, pharmacokinetic profiling and organ biodistribution assays were conducted in xenograft tumor-bearing mice. The siRNA-conjugated DNA tetrahedron nanoarchitecture system was administered to mice by tail vein injection, and the quantitative accumulation was monitored by fluorescence molecular tomography imaging, combined with computed tomography. The imaging data showed accumulation of siRNA-loaded tetrahedrons at the tumor surface 25 min after injection. The siRNA-mediated gene silencing feature was monitored by performing an in vivo firefly luciferase gene expression analysis. The remarkable feature of this study was the one-step synthesis of hybrid DNA–siRNA tetrahedron nanoarchitectures and cation-free gene transfection ability.

### Small-molecule-templated DNA nanoarchitectures

**Figure 4:**
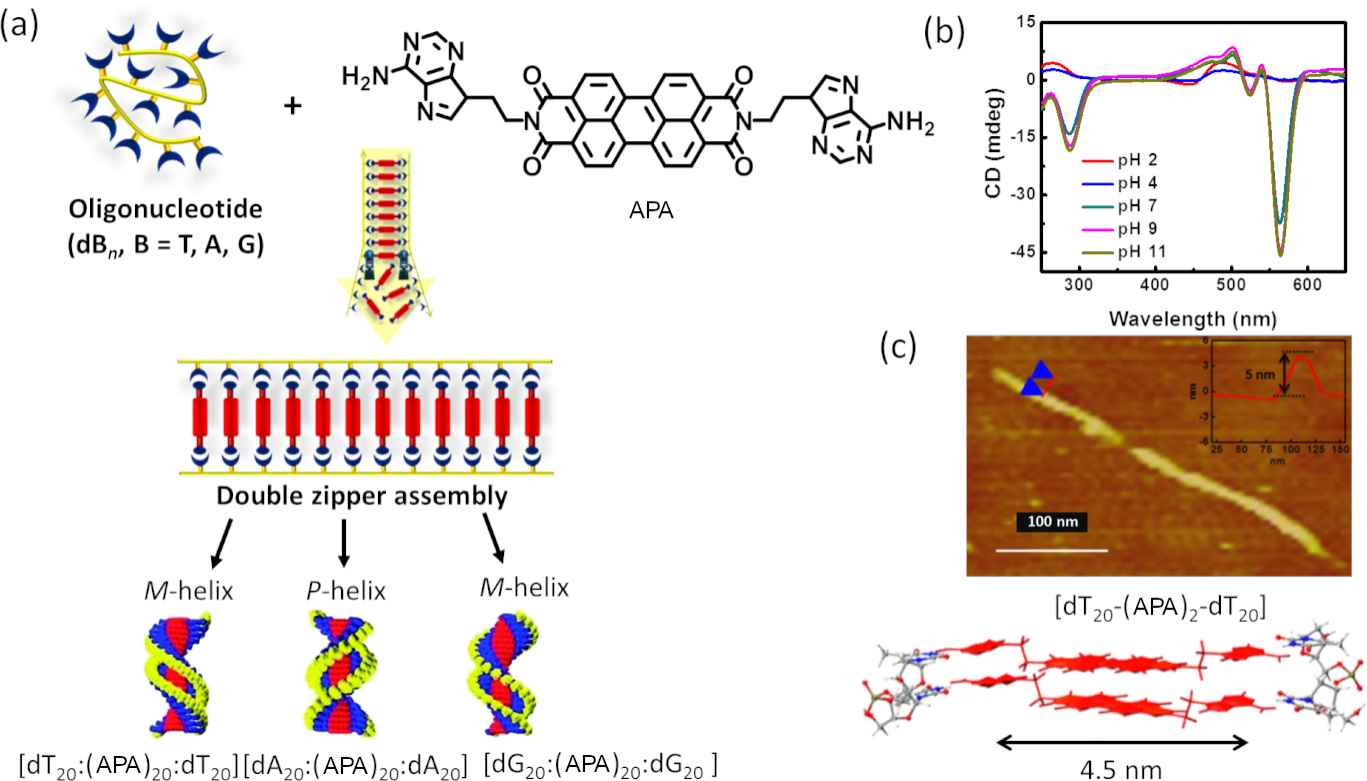
a) Schematic representation of mutually templated double-helical zipper assemblies of APA and dB*_n_* (B: nucleobases T, A, or G; *n* =10, 20) via canonical and noncanonical hydrogen bonding interactions. b) pH-dependent CD measurement of double-helical zipper ensembles. c) AFM image of double-helical zipper assembly nanofiber dT_20_–(APA)_20_–dT_20_ and its height profile data (5 nm, inset), typical thickness 4.5 nm. Figure 4a–c has been adapted with permission from [18], copyright 2015 Royal Society of Chemistry.

The molecular self-assembly of DNA through sequence-specific base pairing is extensively used to create complex nanoarchitectures with variable size and shape in the field of classical DNA nanotechnology. In spite of the possibility of creating complex nanoarchitectures through advanced design and programming, the use of long DNA sequences, high manufacturing cost, and reproducibility of the derived nanoarchitectures are some of the major concerns to be addressed in classical DNA nanotechnology or DNA origami for practical applications. In this context, the emerging field of templated DNA nanotechnology, or functional DNA nanoarchitectonics, is particularly appealing to overcome the trivial drawbacks of DNA nanotechnology in its original form [1,20,25]. The molecular architectonics of SFMs and DNA has enormous potential for the design and construction of SFM-mediated and mutually templated hybrid DNA ensembles and nanoarchitectures with assured functional properties and applications [13,20]. In particular, functional DNA nanoarchitectonic involves the coassembly of suitably designed SFMs and short oligonucleotides supported by canonical and noncanonical hydrogen bonding interactions. Apart from hydrogen bonding, aromatic π–π stacking, electrostatic, metal ion, and host–guest interactions facilitate the molecular coassembly of SFMs and short oligonucleotides. The π-conjugated arylenediimides with interesting optoelectronic properties, such as naphthalenediimide (NDI) and perylenediimide (PDI), are attractive SFMs to support zipper assembly of DNA via π stacking and hydrogen bonding interactions. Our group has successfully demonstrated the molecular architectonics of nucleobase-conjugated arylenediimides and short oligonucleotides through conventional and unconventional base pairing interactions to construct well-defined nanoarchitectures with definite applications [18]. In a unique design, a symmetrically functionalized adenine-conjugated PDI derivative (APA) was designed to interact with thymine via canonical and noncanonical hydrogen bonding, and with guanine via noncanonical hydrogen bonding interactions. The PDI was functionalized with adenine, owing to its unique ability to form hydrogen bonds with other complementary and noncomplementary nucleobases. The canonical hydrogen bonding interaction with oligothymidine resulted in the formation of hybrid DNA ensembles of the type dT*_n_*–(APA)*_n_*–dT*_n_*, *n* = 10, 20, through double-helical zipper assembly (Figure 4a). Further studies revealed that the interaction of APA with dG_10_ and dT_10_ resulted in the formation of M-type double-helical zipper assemblies, while with dA_10_, the formation of a P-type helix was preferred. pH-dependent circular dichroism (CD) measurements showed the collapse of the double-helical zipper assemblies at pH < 6 (Figure 4b). Remarkably, the nanofiber (molecular) structure of the individual double-helical zipper assembly dT_20_–(APA)_20_–dT_20_, extended end-to-end through aromatic interactions, was visualized by AFM (Figure 4c). The left-handedness of the double-helical assembly dT_20_–(APA)_20_–dT_20_ observed in AFM correlated with the CD data. The stimuli-responsiveness of the SFM-supported chirality-imprinted double-helical assembly systems points at potential drug delivery systems for small molecular and gene-based drugs.

**Figure 5:**
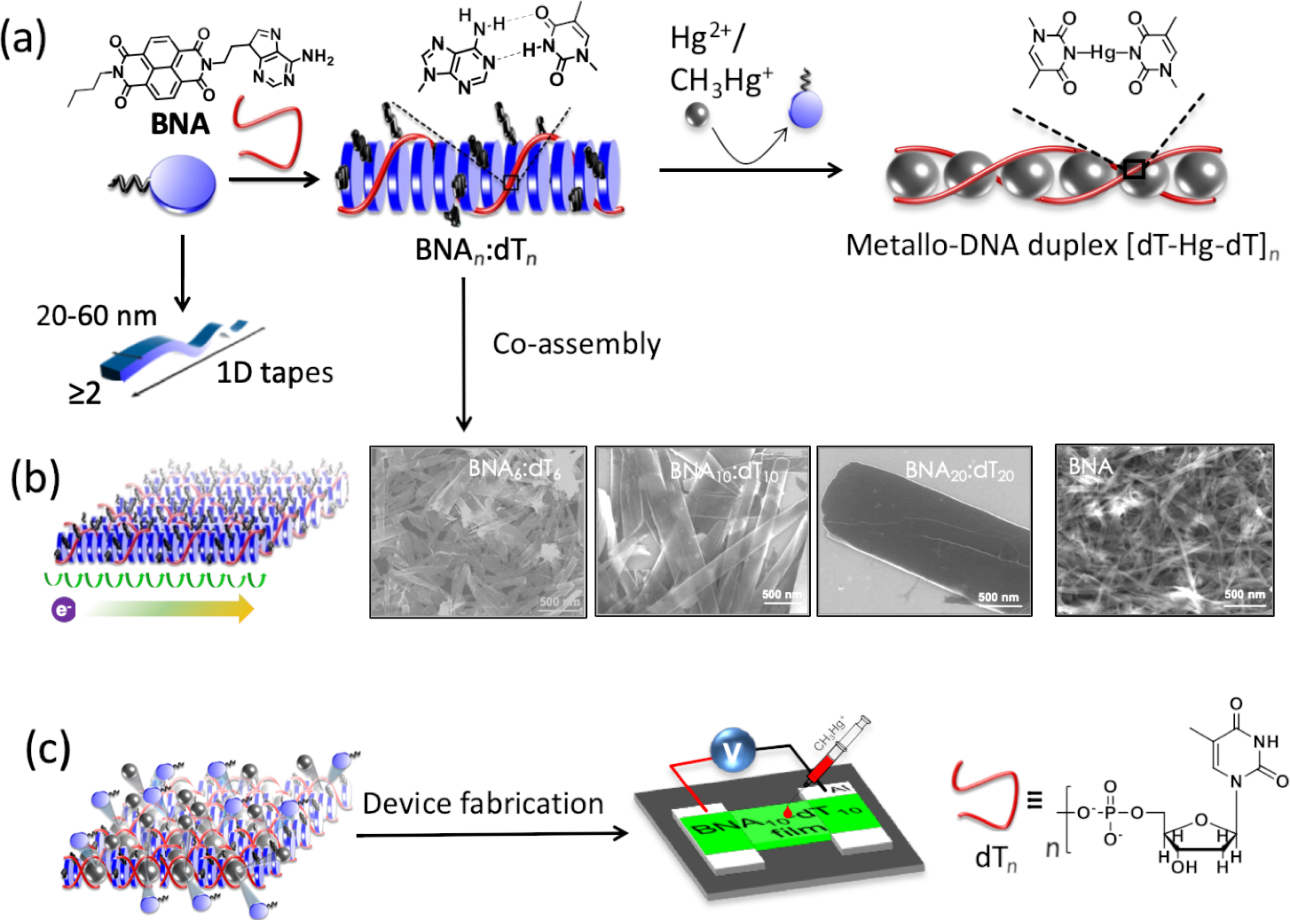
a) Mutually templated coassembly of BNA and dT*_n_* (*n* = 6, 10, 20) to form a BNA*_n_*–dT*_n_* hybrid ensemble, and displacement of BNA from Hg(II), followed by the formation of a metallo-DNA duplex. b) FESEM images of 2D nanoarchitectures (nanosheets) of BNA*n*–dT*n* coassembly and 1D tapes of BNA. c) Schematic representation of a FET device of BNA*_n_*–dT*_n_* fabricated for conductometric sensing of Hg(II) with ultrasensitive sensitivity (0.1 nM, 0.02 ppb). Figure 5a–c has been adapted with permission from [20], copyright 2016 American Chemical Society.

**Figure 6:**
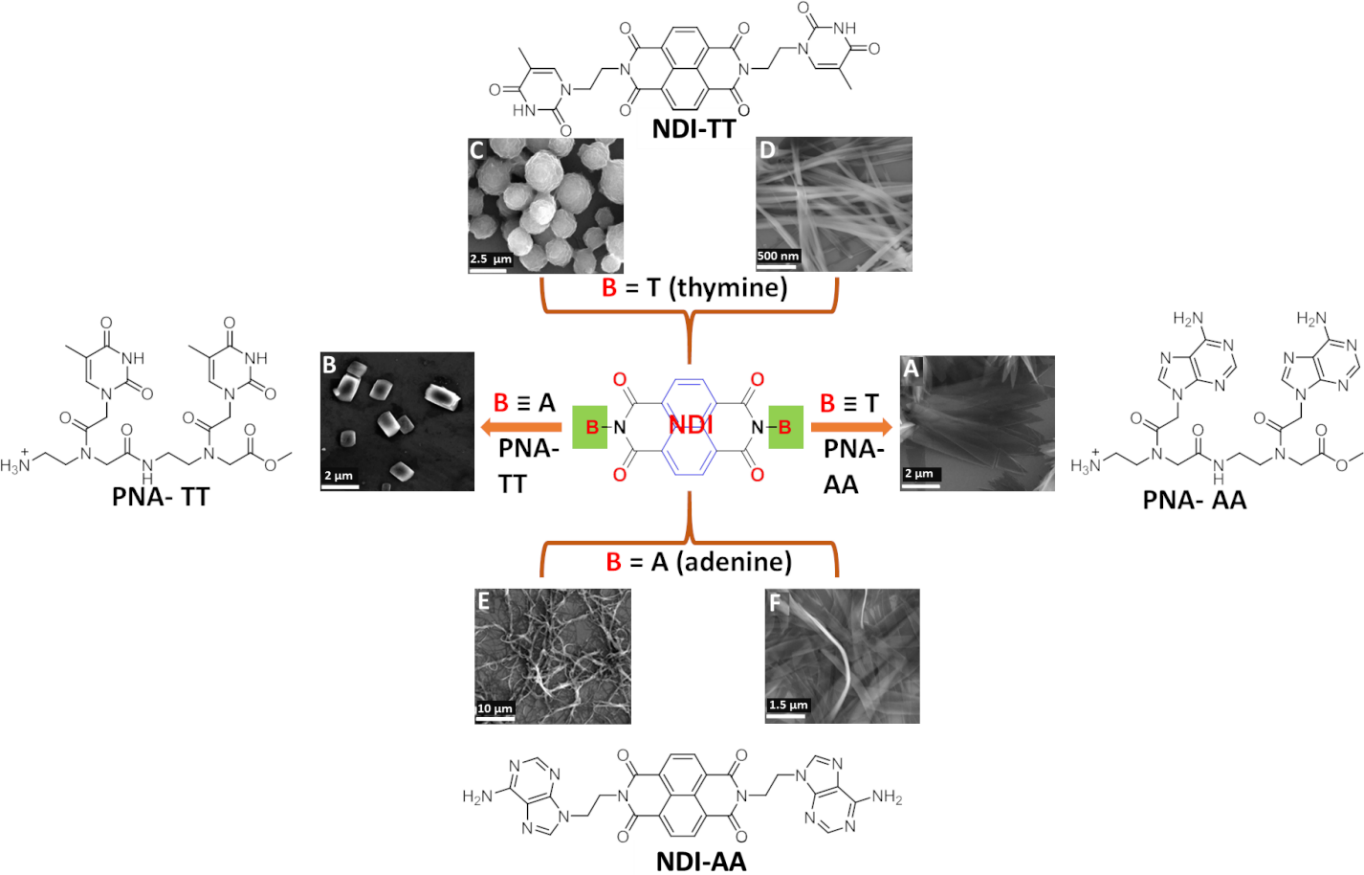
Molecular structures of nucleobase-tethered NDI molecules (NDI-AA and NDI-TT) and their assembly, coassembly, and templated coassembly with PNA clamps. Adapted with permission from [75], copyright 2013 Royal Society of Chemistry.

Mercury is one of the most toxic heavy metals, with a severe impact on human health already at ultralow concentrations [20,72–74]. Selective binding of Hg(II) with thymine is a highly feasible interaction to be exploited for its detection. We utilized the mercury–thymine interaction to develop ultrasensitive detection methods for mercury at subnanomolar concentrations [20]. This design strategy utilizes homothymine (oligothymidine) sequences for the selective and sensitive detection of mercury, and thereby unambiguously circumvents the limitations associated with the earlier reports of DNA-based sensor systems. In our creation strategy, novel DNA nanoarchitectures were designed and developed for the sensing of mercury at subnanomolar levels, wherein adenine-conjugated naphthalenediimide (BNA) forms a mutually templated assembly with oligothymidine (dT*_n_*, *n* = 6, 10, 20, Figure 5a). The BNA*_n_*–dT*_n_* coassembly led to the formation of 2D nanosheets of variable dimensions depending on the dT*_n_* chain length (Figure 5b). Oligothymidine (dT*_n_*) mutually templated with BNA was used for the first time to improve the detection sensitivity for mercury, which involved the thermodynamically and entropically favored displacement of BNA owing to the formation of metallo-DNA duplexes (dT–Hg–dT)*_n_* (Figure 5a). The displacement of BNA caused significant changes in the morphological, chiroptical, and electrical conductivity properties of the system, which was used for the dual-responsive detection of ultralow concentrations (subnanomolar level) of mercury. The BNA*_n_*–dT*_n_* coassembly material was used to fabricate a field-effect transistor (FET) for the detection of mercury (Figure 5c). Remarkably, both chiroptical and conductometric measurement-based data provided subnanomolar detection of mercury (≥0.1 nM, 0.02 ppb), which was 100 times lower than the permitted maximum quantity of mercury in water (≈10 nM, ≈2 ppb), as per the United States Environmental Protection Agency (USEPA). The AFM-based measurement also showed excellent transport properties of individual BNA*_n_*–dT*_n_* nanoarchitectures (nanosheets), which also provided highly sensitive detection of mercury. The displacement of BNA in BNA*_n_*–dT*_n_* by Hg(II) resulted in a change of morphology of the nanoarchitectures from nanosheets to 1D tapes. This unique strategy demonstrated the design and potential application of SFM-supported DNA nanoarchitectures in sensors and bio-optoelectronics. In another study, we reported a molecular architectonic of adenine (A)- and thymine (T)-appended naphthalenediimide derivatives (NDI-AA and NDI-TT, respectively), with peptide nucleic acid (PNA) dimers (clamps) via WC and Hoogsteen base pairing interactions (Figure 6) [75]. The hydrogen bonding interactions, along with hydrophobic interactions, imparted by the nucleobases and the NDI core, facilitated the formation of versatile nano- and microarchitectures. The morphological evaluation showed the formation of petals, fibres, ribbons, and porous spheres by NDI-AA and NDI-TT under different conditions. This study demonstrated the existence of unusual Hoogsteen interactions among the nucleobases that formed 9-membered hydrogen-bonded ring structures instead of an 8-membered WC structure. The chiral ssDNA could host a multitude of diverse molecules and was capable of altering the handedness of the host–guest assembly systems [76]. Switchable helicity was observed when diaminopurine-conjugated naphthalene derivatives interacted as guest molecules within oligothymidine. The helicity of the coassembly systems was found to change with the solutions’ pH value. The reversal of helicity was observed at lower pH, where the right-handed B-DNA form effectively transformed to left handed Z-DNA due to protonation of the guest derivative. Similar experiments performed with adenine and cytosine templates did not show such a reversal of helicity. This report is a typical example for the design of DNA-triggered switchable functional nanoarchitectures.

Reprograming the molecular self-assembly of DNA through noncovalent incorporation of organic molecules can potentially modify and expand the structural diversity and functionality of the resulting nanoarchitectures. In a recent work by Sleiman and co-workers, cyanuric acid (CA) with hydrogen-bonding faces, analogous to three thymine functions, was exploited to modulate the assembly of ssDNA to form unique nanofiber architectures [77]. The novelty of this work lies in the use of a small molecule, CA, that promoted the self-assembly-driven reprograming of polyadenine strands into a noncanonical motif. The interactions between adenine and CA were consistent with the formation of hexameric rosette architectures, and CA facilitated the cooperative growth of polyadenine strands with a very high aspect ratio.

The secondary structure of biomolecules depends on weak noncovalent interactions, especially ionic interactions, between oppositely charged moieties or surfaces. In fact, ionic or electrostatic interactions can play an important role in the scheme of templated DNA nanoarchitectonics. Similarly, fundamental DNA binding interactions of small molecules, viz., intercalation and groove binding, can be used to construct small molecule–DNA ensembles. Williams and co-workers reported a distinct binding mechanism based on the so-called phosphate clamp, wherein the DNA backbone phosphate groups were used for the interaction with small functional molecules [78]. Essentially, the interaction of cationic small molecules with the anionic periphery of DNA led to the formation of electrostatically stable small molecule–DNA ensembles. The aromatic molecules with cationic functionalities were capable of imparting dual stabilization through π stacking and electrostatic interactions. Ulrich and co-workers reported the interaction of aromatic molecules bisfunctionalized with guanidinium moieties and ssDNA in aqueous solution [79]. The cationic guanidinium moieties interacted with the anionic phosphodiester backbone of DNA, while aromatic π stacking played a significant role in the molecular assembly in aqueous solution. In another study, isophthalamide and dipicolinamide molecules were shown to act as synthetic small-molecule vectors for the transfection of plasmid DNA [80]. The dipicolinamide molecules acted as an anion (phosphate) binder and exhibited channel forming properties, thereby becoming an efficient biomaterial for the binding and delivery of cargo DNA. *Escherichia coli* was chosen as representative bacterium for transfection studies. The dipicolinamide-guided transfection with plasmid DNA was found to stimulate the growth of *E. coli*, which confirmed the good transfection efficiency of the small-molecule (dipicolinamide) vector.

Izawa and co-workers reported the use of anthracene derivatives to drive the self-assembly of ssDNA into helical nanofibers [81]. The weak interactions between thymidylic acid-conjugated anthracenes and complementary oligoadenylic acid resulted in the formation of helical J-aggregates via A–T base pairing interactions. The characterizations performed using spectroscopic and microscopic methods suggested the binary self-assembly between the anthracene derivatives and 20-meric oligodeoxyadenylic acid where the transition dipolar axis of the anthracene derivatives was aligned in a head-to-tail fashion. The UV–vis and CD spectroscopy data showed cooperative changes in the binary self-assembly in response to the temperature. Shimizu and co-workers reported the synthesis of nucleotide-tethered oligo(*p*-phenylene vinylene), (2,1-ethenediyl-1,4-phenylenemethylene)bis(2′-deoxy-3′-thymidylic acid), and examined the complementary interaction with 20-meric oligodeoxyadenylic acid in water [82]. The binary self-assembly between the oligo(*p*-phenylene vinylene) and oligodeoxyadenylic acid in water resulted in the formation of right-handed helical stacks of different diameters, based on the residual stoichiometry of the two components. However, the interaction with noncomplementary 20-meric oligothymidylic acid did not produce any self-assembled structure in water. In another study, the interaction between a thymidine bolaamphiphile dTp–20–dTp and a series of oligoadenylic acids d(A)*_n_* (*n* = 2, 4, 6, 8, 10, 20, 40) was found to form nanofibers with a double-helical structure [83]. The binary self-assembly interaction between the bolaamphiphile and the oligoadenylic acids dTp–20–dTp/d(A)*_n_*, *n* = 2, 4, 6, 8, 10, 20, 40, strictly depended on the chain length of the oligoadenylic acid. Through the presence of equal amounts of adenine and thymine within the ensembles, it was possible to form hydrogels in water upon incubation for several days. However, an increase in oligoadenylic acid chain length resulted in changes in hydrogel color and rigidity.

### Metal–base pair interactions-guided design of DNA nanoarchitectures

**Figure 7:**
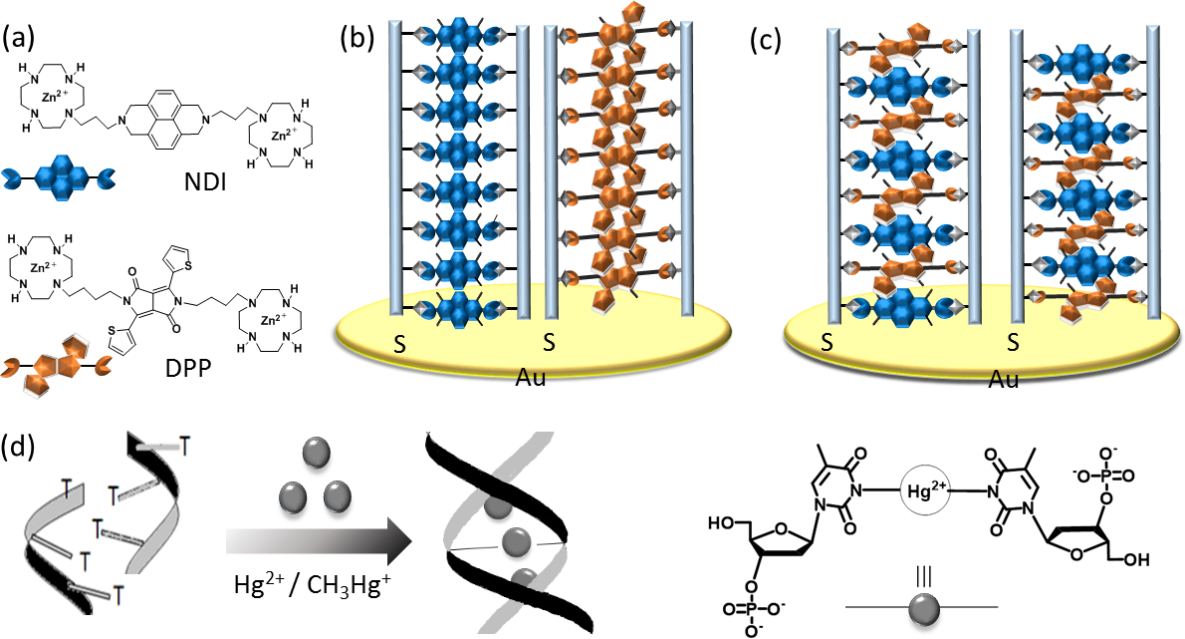
a) Zn(II)-cyclen-tethered NDI and DPP SFMs. b) DPP–dT_40_ and NDI–dT_40_ multichromophore arrays over a gold electrode via coimmobilizing donor–acceptor units. c) Random assembly of a DPP–NDI–dT_40_ multichromophore array over a gold electrode. d) Formation of a metallo-DNA duplex through T–Hg(II)–T interactions, maintaining the 2:1 molar ratio of T and Hg(II). Figure 7a–c has been adapted with permission from [84], copyright 2015 Wiley and Sons, and Figure 7d has been adapted from [86], copyright 2006 American Chemical Society.

Metal–base pair interaction-driven molecular architectonics are one of the major alternatives to hydrogen bonding (WC and non-WC)-supported base pair interactions for the development of functional DNA nanoarchitectures. Nakamura and co-workers reported the construction of metal–ssDNA coassembly systems wherein Zn(II)-bis(cyclen)-conjugated NDI and diketopyrrolopyrrole (DPP)-based multichromophore units were used for mutual templating of short oligonucleotides (Figure 7a) [84]. In their previous reports, Zn(II)-bis(cyclen)-conjugated NDI were shown to guide the formation of multichromophore arrays via binding with dT*_n_*, and the lengths of the multichromophore arrays were found to be dependent on the dT*_n_* chain length and temperature [85]. The multichromophoric array of NDI and oligo-dT*_n_* assembled over a gold substrate showed photocurrent generation due to electron conduction because of the π‐stacked array of the NDI assembly. In their next design approach, two separate zinc binding systems, Zn(II)-bis(cyclen)–NDI and Zn(II)-bis(cyclen)–DPP, were decorated using oligothymidine (dT_40_) as a scaffold via interaction of Zn(II)-bis(cyclen) with thymidine residues. The ensemble behaved as a donor–acceptor heterojunction system where DPP acted as a donor moiety and NDI as an acceptor moiety. One of the advantages of this system was that the DNA–multichromophore organization could be aligned vertically over the gold electrode, which facilitated exothermic charge separation and suppressesed the ground-state charge transfer (CT) complexation between DPP and NDI, followed by the generation of a photocurrent (Figure 7b). However, the randomly assembled array of DPPNDI–dT_40_ immobilized across the gold electrode was unable to generate any photocurrent response owing to ground-state CT complexation of DPP with NDI in their random arrangement (Figure 7c). Tanaka, Ono, and co-workers reported the generation of mercury-mediated base pairing T–Hg(II)–T band metallo-DNA duplex structures (Figure 7d) [86]. Lu and co-workers exploited the T–Hg(II)–T metal–base pairing to control the DNAzyme activity through allosteric interactions [87]. For the first time, we showed the mercury-mediated displacement of SFM (BNA) from the BNA_n_–dT*_n_* hybrid ensemble to form a metallo-DNA duplex of homothymidine sequences, and this transformation was used as a chiroptical and conductometric sensor platform for the ultrasensitive detection of mercury at a subnanomolar level (vide supra) [20,72]. The remarkable outcome of our design strategy was attributed to the ultrasensitive detection of mercury through FET device fabrication, which overcame the limitations of earlier reports on DNA-based Hg(II) detection.

### Chromophore conjugation-guided DNA architectonics

**Figure 8:**
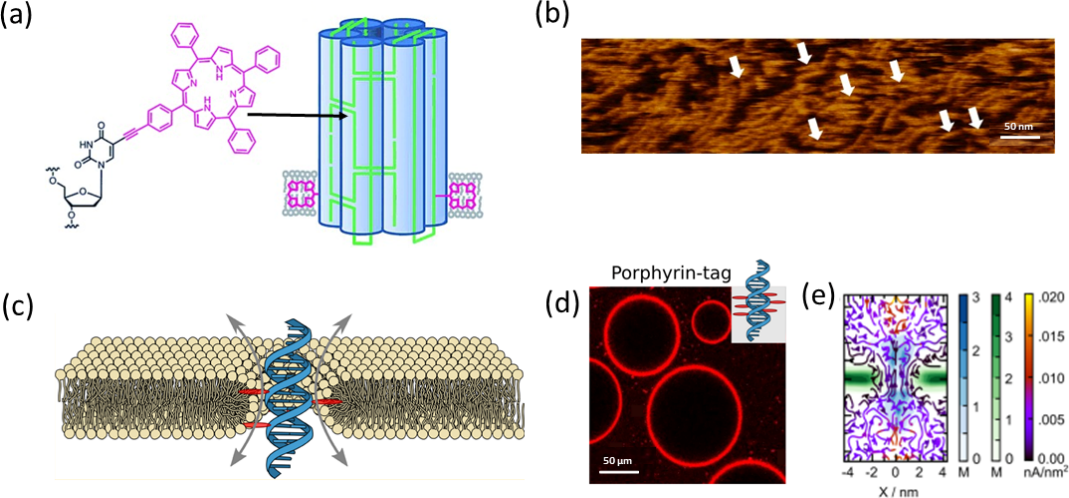
a) Schematic representation of a porphyrin-appended DNA nanopore base lipid anchor. b) AFM image of a nanopore assembly. c) Schematic design of a membrane-spanning porphyrin-tagged DNA duplex. d) Fluorescence confocal image highlighting the interaction of porphyrin-tethered DNA with lipid membrane. e) Molecular dynamics simulation to analyze steady-state local densities of porphyrin–DNA-anchored lipid chains and their current flow. Figure 8a and Figure 8b were adapted from [93], distributed under a Creative Commons Attribution license, copyright 2013 by the authors. Figure 8c–e was adapted with permission from [94], copyright 2016 American Chemical Society.

The introduction of organic chromophores within the nucleic acid system is one of the distinct approaches to generate functional DNA architectures [88]. Porphyrins are well-known macrocyclic organic chromophores acting as light harvesting systems that can be efficiently compacted within the spatial arrangements of DNA double-helical assemblies [88]. Meunier [89], Hélène [90], and co-workers reported the utility of porphyrin-tethered DNA as artificial nucleases. Murashima, Sugimoto, and co-workers adopted a novel approach to design DNA nanoarchitectures by substituting the nucleobases of DNA with porphyrins [91]. The tetraphenylporphyrin-modified nucleotide was inserted into the center of a 13-mer oligonucleotide sequence in an automated DNA synthesizer through phosphoramidite chemistry. The annealing of porphyrin-tethered oligonucleotides with complementary oligonucleotides resulted in the formation of a B-form DNA duplex. The conformational distortion effect due to the intercalation of porphyrin was neutralized via stabilization of the ensemble by stacking interactions that created the B-form duplex structure. Sitaula, Reed, and co-workers reported the ligation of a porphyrin derivative by a 19-nucleotide DNA sequence [92]. The porphyrin units were ligated by DNA via direct amidation, and the covalent attachment allowed the insertion of an array of porphyrin segments along the nucleotide sequence. Recently, a transmembrane lipid bilayer nanopore comprised of folded DNA became the center of attraction by mimicking natural protein pores. Howorka and co-workers reported the synthesis of porphyrin-conjugated DNA nanopores as a simple and effective strategy to span through the bilayer system [93]. The nanopore consisted of six hexagonally packed DNA double-helical assemblies that were preserved by double-crossover strands (Figure 8a). The two porphyrin units were positioned at the terminal of a helical bundle that improved the directional insertion of the nanobarrel across the bilayer. AFM images showed that the assembled morphology of the hexagonally packed nanobarrels was made up of porphyrin-tethered DNA (Figure 8b). Stulz and co-workers designed a porphyrin-tethered single-DNA duplex as a transmembrane ion channel [94]. Their minimalistic design approach involved the attachment of six porphyrin units along the oligonucleotide sequence that facilitated the movement of ions through the channel. The schematic of molecular dynamics simulation data (Figure 8c) showed the movement of ions through the lipid–nanopore interface via the formation of a toroidal pore. The binding of porphyrin-tethered nanopores with giant unilamellar vesicles was analyzed by confocal microscopy (Figure 8d). The inherent fluorescent signal of porphyrin showed their presence across the lipid vesicle. Further investigations on ionic current traces demonstrated that the lipid membrane insertion and gating behavior of the nanopore resembled natural protein channels (Figure 8e). Embodiment of the porphyrin within the DNA system played dual roles, viz., imparting hydrophobic effects and characteristic optical responses to monitor the insertion mechanism. Notably, the position of conjugation and the number of porphyrin units were crucial parameters that significantly affected the insertion process. Asanuma and co-workers incorporated six methyl red chromophores into a double-helical DNA through ᴅ- and ʟ-threolinol linkers to analyze the molecular exciton theory of heterodimeric chromophores [95]. NMR studies revealed the antiparallel orientation of the two dyes across the duplex strand. Further studies indicated that the increment in dye number could dramatically affect the spectroscopic behavior and the solution properties of the dye.

### DNA–metal nanoparticle architectures

Interactions of DNA with nanoparticles are an attractive area for the fabrication of functional DNA nanoarchitectures [96]. The surface properties of the nanoparticles are greatly influenced by the functionalization with DNA. The crystallization of nanoparticles can be easily programed by the selection of designer DNA sequences. In fact, DNA-mediated assembly is widely used to synthesize hybrid lattices of gold nanoparticles and protein-based capsid particles [97–98]. The methylation of DNA is one of the epigenetic modifications that include the addition of a methyl group to cytosine, and this modification controls the genetic programing in living system. The epigenetically reprogrammed methylation landscape in cells is a marker for several types of cancer. The cancer pathology is characterized by different methylation patterns, which include the net loss of global methylation throughout the genome sequence, while the regulatory or promoter regions of DNA involve a high rate of methylation. The differences in genomic distribution of methylation greatly influence the solution properties of DNA, and this concept has been exploited by Trau and co-workers to analyze the different interaction patterns of methylated and nonmethylated DNA with gold nanoparticles [22]. The consistent and high methylation level of genomes makes the DNA more hydrophobic, while the distinct methylation in cancer genomes shows much lower hydrophobicity. The changes in solvation properties of DNA drastically affect the affinity towards metal nanoparticles. Based on these aspects, the authors have efficiently evaluated the affinity of genomic DNA towards metal nanoparticles depending on the methylation pattern and extent of methylation throughout the genome. In particular, this constituted a promising sensor-free diagnostic platform for the detection of cancer. Chen and co-workers showed that the interaction of DNA with dithiothreitol (DTT)‐conjugated gold nanoclusters (Au NCs) results in the formation of ‘raspberry‐like’ particles with potential gene delivery application [99]. The novelty of the ‘raspberry-like’ structures lay in their biocompatible ‘shield’, which protected the capped DNA from enzymatic degradation. Gianneschi and co-workers documented the synthesis of crystalline gold nanowires from DNA block copolymer micelles [100]. The block copolymer micelles were synthesized through ring-opening polymerization, followed by the integration of DNA oligonucleotides in a postpolymerization modification process. The DNA acted as a polar head group, while the whole polymer system acted as a template for nanowire synthesis. The miniaturization of devices is of central importance in electronics and has galvanized significant research in materials science. The need for the miniaturization of devices at the nanoscale interface has led to the exploration of new material systems and building blocks, while relinquishing the use of traditional silicon-based materials also played a key role. Biomolecular nanolithography is a newer approach to create nanopatterned surfaces using biomolecules as scaffolds [101–102]. The interesting features of this technique are the combination of two separate scales, the nanoscale and the biomacromolecular interface, to produce functional architectures. In this context, the well-defined structural organization, anionic properties, and polymeric nature qualify DNA and RNA as potential candidates for scaffolding purposes. Employing the nanolithography technique, precise DNA nanostructures can be constructed, and these DNA nanoarchitectures exhibit remarkable molecular recognition properties at the nanoscale. Hutchison and co-workers reported the use of DNA as a scaffold for nanoscale patterning of metal nanoparticles over a DNA surface [103]. The electrostatic interaction with the ligand stabilized the metal nanoparticles, and the backbone phosphate groups of DNA triggered the formation of different nanoarchitectures, such as ribbons, linear chains, and branched structures. Patolsky and co-workers documented the photochemical covalent attachment of gold nanoparticles to DNA surfaces, which resulted in the formation of a gold nanowire [104]. The synthesis of DNA gold nanowires was followed by the incorporation of psoralen‐functionalized gold nanoparticles across double-stranded DNA. Psoralen acted as an intercalator that led to photochemical crosslinking of the components within the DNA matrix. The photoinduced [2+2] cycloaddition of the thymine residues of DNA in the psoralen‐functionalized gold nanoparticle matrix resulted in the formation of conductive nanorings or nanowires. Willner and co-workers reported a novel nanoarchitectonic consisting of a DNA-crosslinked CdS nanoparticle array on an electrode surface that generated an efficient photocurrent upon irradiation [105]. The electrostatic interaction of [Ru(NH_3_)_6_]^3+^ with the DNA-bound CdS nanoparticle system supported tunneling of the conduction band electrons, followed by the generation of photocurrents. Similarly, λ‐DNA was used as a biogenic template for the generation of silver nanowires and the synthesis of palladium nanoparticle clusters [106–107].

## Conclusion and Outlook

The disruptive idea of using DNA as a material building block (DNA nanotechnology) has brought a revolution to the broad areas of nanoscience and nanotechnology. A large number of research groups have been involved in the rational design of DNA-based nanomaterials, origami, nanomachines, and devices. The unique structural features enabled by predictable sequence-specific interactions have made DNA a versatile component for programmable molecular architectonics to construct diverse molecular and nanoarchitectures. In DNA nanotechnology, robust design and programming protocols have led to the creation of 1D, 2D, and 3D nanoarchitectures using well-defined tiles and bricks. Remarkably, complex DNA architectures in terms of shape and size could be achieved through the concept of DNA origami. While DNA nanotechnology (and origami) is extremely creative in its original form and produced complex nanoarchitectures of virtually any shape and size through standard and robust DNA self-assembly, potential applications of these materials are yet be realized. In this context, templated DNA nanotechnology or functional DNA nanoarchitectonics have been conceived to overcome the limitations of classical DNA nanotechnology. In this simple and novel approach, molecular architectonics of suitably tethered SFMs, or their assemblies, and ssDNA produced hybrid DNA ensembles with functional properties and practical applications. Mutually templated coassemblies of SFMs and ssDNA were orchestrated through conventional (WC) and unconventional (non-WC) hydrogen bonding-, aromatic π–π stacking-, electrostatic-, and metal coordination-based interactions. Notably, novel functional DNA nanoarchitectures were produced through the coassembly of inexpensive short ssDNA sequences (oligonucleotides) and SFMs that ensured practical applications. The solution-based molecular architectonics approach to design and construct SFM-tethered hybrid DNA ensembles opened up new possibilities in the fields of materials science, nanoscience, nanotechnology, and biomedicine. Organic chemistry played a key role in the design and synthesis of SFMs suitable for the coassembly with ssDNA to cater for specific applications, ranging from novel materials design, optoelectronics, sensors, diagnostics, and drug delivery to therapy. The outlook for the emerging field of functional DNA nanoarchitectonics lies in the controlled and programmed coassembly (molecular architectonics) through mutual templating of SFMs or other designer units and ssDNA to generate novel molecular and nanoarchitectures with emergent properties and practical applications in the domains of health, energy, and environment.
